# Association between fibroblast growth factor 23 and functional capacity among independent elderly individuals

**DOI:** 10.31744/einstein_journal/2021AO5925

**Published:** 2021-07-22

**Authors:** Lara Miguel Quirino Araújo, Patrícia Ferreira do Prado Moreira, Clineu de Mello Almada, Luciano Vieira de Araújo, Aline Granja Costa, Ricardo de Castro Cintra Sesso, John P Bilezikian, Marise Lazaretti-Castro, Maysa Seabra Cendoroglo

**Affiliations:** 1 Escola Paulista de Medicina Universidade Federal de São Paulo São PauloSP Brazil Escola Paulista de Medicina, Universidade Federal de São Paulo, São Paulo, SP, Brazil.; 2 Escola de Artes, Ciências e Humanidades Universidade de São Paulo São PauloSP Brazil Escola de Artes, Ciências e Humanidades, Universidade de São Paulo, São Paulo, SP, Brazil.; 3 Department of Endocrinology College of Physicians and Surgeons Columbia University Nova IorqueNY United States Department of Endocrinology, College of Physicians and Surgeons, Columbia University, Nova Iorque, NY, United States.

**Keywords:** Fibroblast growth factors, Functional status, Cognitive aging, Aging, Aged, 80 and over, Kidney/physiology, Activities of daily living, Sarcopenia

## Abstract

**Objective:**

To examine the association of between serum fibroblast growth factor 23 and the functional capacity among independent individuals, aged 80 or older.

**Methods:**

The functional capacity of 144 elderly was assessed by Instrumental Activities of Daily Living, cognitive tests, handgrip strength and the timed ability to rise from a chair and sit down five times. Fibroblast growth factor 23 was measured using an ELISA assay.

**Results:**

Participants in the lowest fibroblast growth factor 23 tertile had the highest mean±standard deviation estimated glomerular filtration rate, the highest mean hemoglobin level, the lowest average number of diseases and the lowest number of medications used. In participants with the estimated glomerular filtration rate >45mL/minute/1.73m^2^, mean fibroblast growth factor 23 level was higher in those with 25(OH) vitamin D <20ng/mL than in those with 25(OH) vitamin D ≥20ng/mL (75.6RU/mL±42.8 *versus* 68.5RU/mL±41.7; p<0.001). There was an increase in the mean serum cystatin C (from 1.3mg/mL±0.3 to 1.5mg/mL±0.3 to 1.7mg/mL±0.4) as function of higher fibroblast growth factor 23 tertile (p<0.001). Fibroblast growth factor 23 levels were not significantly associated with capacity in physical or cognitive tests.

**Conclusion:**

In independent community-dwelling elderly, aged ≥80 years, fibroblast growth factor 23 was associated with aged-related comorbidities and renal function but not with functional capacity.

## INTRODUCTION

The identification of biomarkers of the aging process has the potential to promote the development of targets to prevent aging-associated syndromes. Fibroblast growth factor 23 (FGF23) is a candidate target because it is a key protein in aging-associated biochemical pathways. The FGF-Klotho endocrine axes plays a decisive role in aging-related diseases. Fibroblast growth factor 23 is an endocrine protein that enter in systemic circulation and the Klotho protein is the co-receptor to bind FGF23, with high affinity to fibroblast growth factor receptor (FGFR) in their target organs. The kidneys are the FGF23’s major target organ, where it induces phosphaturia by acting in the apical brush border membrane of proximal tubular cells. Moreover, FGF23, vitamin D, and parathyroid hormone (PTH) are in negative feedback loops to maintain phosphate homeostasis.^(1)^

The lack of FGF23 or Klotho causes a premature aging phenotype, such as hypogonadism, premature thymic involution, ectopic calcification, dermal atrophy, pulmonary emphysema, neurodegeneration, hearing loss and vascular calcifications.^(2-5)^ There is some evidence to support this aging phenotype based on hyperphosphatemia.^(6)^ In chronic kidney disease (CKD) and in the aging kidney, FGF23 serum level rises to control hyperphosphatemia via the FGF23-Klotho-vitamin D-PTH axis. However, FGF23 fails to maintain phosphate balance, leading to hyperphosphatasemia, and the phosphate starts to exert its deleterious actions. On the other hand, an experiment with human muscle mesenchymal stem cells, important in the regeneration of muscle mass, showed that treatment with FGF23 promotes the aging phenotype independently of Klotho, through direct action in the regulation of cell cycle by p53 protein.^(7)^ Paradoxically, an experiment with physical exercise protocols in mice demonstrated an increase in serum FGF23 and longer-term exercise upregulated FGF23 mRNA and FGF23 in skeletal muscle. And the mice previously treated with FGF23 performed better in exercise, control of reactive oxidative species (ROS), and improved mitochondrial function in skeletal muscle. The mechanism for these actions is unknown.^(8)^ These studies suggest that the influence of FGF23 on muscle biology and physical performance depends on the context and different pathways. *Deficit* of FGF23 in serum causes premature aging by loosing of phosphate control and FGF23 overload can lead to cell senescence by p53. We could speculate that accelerated aging in CKD may be influenced by both mechanisms, depending on CDK stage. Conversely, serum FGF23 elevated by a healthy stimulus, such as physical exercise, could improve performance in physical exercise.

The health of elderly individuals should be understood from a functional perspective (and not based on the disease), since healthy aging is “the process of developing and maintaining the functional ability that enables wellbeing in older age”. Functional capacity means “the health-related attributes that enable people to be and to do what they have reason to value”.^(9)^ Cognitive function and physical performance are pillars of these attributes and FGF23 may influence both. A sample from the Framingham Heart Study showed an association of serum FGF23 levels and incidence of dementia,^(10)^ and FGF23’s actions in muscle biology and premature aging may play a role in physical performance.

## OBJECTIVE

To examine the association between serum fibroblast growth factor 23 and functional capacity among independent individuals aged 80 or over, as well as to investigated the association of fibroblast growth factor 23 with laboratory measurements of kidney-vitamin D-parathyroid hormone axis.

## METHODS

### Study participants

This is a cross sectional study of data from the first wave of the Longevity Project, a cohort of community-dwelling elderly aged 80 years or more, independent for activities of daily life,^(11)^ and recruited by advertisement at local neighborhood newspapers, radio media and referral from other departments of the *Universidade Federal de São Paulo* (UNIFESP) to the Geriatrics Division, from 2010 to 2012. The inclusion criteria were to be able to walk without human support, to have all chronic disease under control at the time of the study, and no cognitive complains. The exclusion criteria were living in a nursing home, apparent cognitive impairment, end-stage disease, being unable to respond to the study tests, apparent infection, heart failure, liver cirrhosis, history of dialysis, immunosuppressive therapy in 6 months, previous chemotherapy for cancer, or HIV infection. All signed the Informed Consent Form, and the Research Ethics Committee of UNIFESP approved the study protocol 1532/09.

### Measurements

Sociodemographic data and body mass index (BMI), clinical data, medication inventory and functional capacity domains were collected using a standard questionnaire administered by trained physicians.

### Functional capacity

Depressive symptoms were obtained using the 15-item Geriatric Depression Scale.^(12)^ Cognitive function was assessed by the Mini-Mental State Examination (MMSE)^(13)^ and by category Verbal Fluency Test (VFT).^(14)^ The MMSE is a test of global cognition and the VFT tests language, executive functions and semantic memory. The self-reported ability to perform tasks for life in the community was assessed using Lawton’s scale of Instrumental Activities of Daily Living (IADL), scored from seven to 21 (the highest score means independence).^(15)^Physical performance was assessed by the timed ability to rise from a chair and sit down five times,^(16)^ and handgrip strength by the best result out of three attempts on a Jamar dynamometer.^(17)^

### Laboratory measurements

Anthropometric and performance measurements were performed at the same time, and blood was collected in the following 4 months. The blood samples were drawn after a 12 hours fast, and creatinine, cystatine C, hemoglobin, albumin, serum 25(OH) vitamin D and intact PTH levels were measured. Serum FGF23 was analyzed by a second generation C-terminal FGF23 two-site Enzyme Linked Immunonosorbent Assay (ELISA; Immutopics, San Clemente, CA, USA). This assay detects two epitopes in the C-terminus of FGF23, and has a sensitivity of 1.5 relative units per mL (RU/mL), and inter- and intra-assay coefficients of variations of less than 5%. Serum creatinine was measured using a modified kinetic Jaffé colorimetric method on an autoanalyzer (AU 400, Beckman Coulter, CA. USA), which was calibrated to isotope dilution mass spectrometry, using a standard reference material (914a) traceable to the National Institutes of Standards and Technology (NIST). Plasma cystatin C levels were determined by an automated particle-enhanced immunoturbidimetric method using a Beckman AU 400 analyzer (Beckman Coulter), and reagents (code number LX002. s2361. X0973. X0974) obtained from DakoCytomation (Glostrup, Denmark), following the procedures recommended by the reagent producer. We estimated glomerular filtration rate (eGFR) by the CKD-EPI creatinine-cystatin C equation. All samples were frozen at −80°C.

### Data analyses

The analyses were performed using the (SPSS) version 20.0. We categorized FGF23 levels into tertiles and examined the distribution of covariates in their categories. The functional capacity domains were presented as mean (±standard deviation – SD). We used the χ^2^ test to examine the associations between the categorical variables; the analysis of variance (Anova) was used to compare the means of more than two groups; the Duncan test or the Dunnett’s C test we used for multiple comparisons; the Kolmogorov-Smirnov test and Levene test were used to verify the normality and homoscedasticity, respectively; the Brown-Forsythe correction was employed in case of mistakenly presuming homoscedasticity to correct the degrees of freedom of the F statistics; the Kruskal-Wallis was used in case of non-parametric distribution; and if the Kruskal-Wallis test was significant, the difference between groups was assessed using the Bonferroni-Dunn. In the multiple linear regression, clinical characteristics were independent variables and FGF23 was a dependent variable. Initially all variables were included in the model, then a non-significant 5% were excluded one by one in order of significance (backward method).

## RESULTS

We studied 144 patients, with mean age of 85.4 years, 73.6% were women, with an average of four chronic diseases and regular use of six medications. Overall FGF23 was 91.05RU/mL±67.47. The eGFR less than 45mL/minute/1.73m^2^was noted in 33.3%, and 25(OH) vitamin D <20ng/mL was present in 59% of all participants. Table 1 shows all participants grouped by FGF23 tertiles to investigate demographic, clinical, laboratory and functional capacity differences.

**Table 1:** Demographic, clinical, and laboratory parameters of the study population by tertiles of fibroblast growth factor 23 levels

Characteristics	All sample (n=144)	FGF23 (RU/mL)			p value
		1^st^ tertile <54.4 (n=47)	2^nd^ tertile 54.4-92.6 (n=48)	3^rd^ tertile ≥92.6 (n=49)	
Age, years	85.4±4.1	85.6±3.8	84.1±3.0^a^	86.6±4.9^b^	0.011
Gender					0.931
Male	38 (26.4)	13 (27.7)	13 (27.1)	12 (24.5)	
Female	106 (73.6)	34 (72.3)	35 (72.9)	37 (75.5)	
Race					0.069
White	96 (66.7)	26 (55.3)	32 (66.7)	38 (77.6)	
Non-white	48 (33.3)	21 (44.7)	16 (33.3)	11 (22.4)	
Number of chronic disease	4.4±1.9	3.9±1.9^a^	4.5±1.6	4.9±2.0^b^	0.018
Number of regular use medications	5.9±2.7	4.9±2.1^a^	6.2±2.6^b^	6.5±3.1^b^	0.008
Vitamin D supplements	59 (41.3)	15 (31.9)	23 (47.9)	21 (43.8)	0.260
Weight, kg	70.7±78.9	65.1±12.5	82.4±135.6	64.7±13.8	0.687
Body mass index, kg/m^2^	26.8±4.3	27.4±4.0	26.5±4.1	26.5±4.9	0.562
25(OH)D, ng/mL	19.2±8.4	19.6±8.6	20.8±9.6	17.3±6.4	0.110
25(OH)D – category, ng/mL					0.183
<20ng/mL	85 (59.0)	26 (55.3)	25 (52.1)	34 (69.4)	
≥20ng/mL	59 (41.0)	21 (44.7)	23 (47.9)	15 (30.6)	
PTH, pg/mL	62.6±33.4	53.9±29.2	63.1±27.8	70.5±40.0	0.060*
Calcium, mg/dL	9.3±0.5	9.3±0.5	9.4±0.4	9.4±0.5	0.428
Phosphorus, mg/dL	3.4±1.1	3.2±0.5	3.3±0.4	3.7±1.8	0.091*
Hemoglobin, g/dL	13.4±1.3	13.9±1.2^b^	13.3±1.3^a^	13.1±1.3^a^	0.012
Creatinine, mg/dL	0.96±0.28	0.87±0.19^a^	0.97±0.24^b^	1.04±0.35^b^	0.007
Cystatin C, mg/L	1.50±0.37	1.32±0.28^c^	1.49±0.29^a^	1.69±0.43^b^	<0.001
eGFR, mL/minute/1.73m^2^	51±14	58±12^b^	50±12^a^	45±14^a^	<0.001
eGFR – category, mL/minute/1.73 m^2^					<0.001
<45mL/minute/1.73m^2^	48 (33.3)	6 (12.8)^a^	17 (35.4)	25 (51.0)^b^	
≥45mL/minute/1.73m^2^	96 (66.7)	41 (87.2)	31 (64.6)	24 (49.0)	
Functional capacity					
Education, years	4.5±3.8	4.4±3.7	5.1±4.6	3.9±2.9	0.720*
GDS	3.9±2.7	3.4±2.2	3.9±2.9	4.4±2.8	0.259*
MMSE	24.2±3.4	23.7±3.2	24.5±3.6	24.5±3.3	0.466
VFT	12.9±3.5	12.5±3.4	12.9±3.6	13.3±3.7	0.562
IADL	18.8±2.5	19.0±2.6	19.3±2.1	18.2±2.8	0.156*
Handgrip strength, kgf^†^					
All	21.8±9.0	23.5±12.4	21.6±6.3	20.5±7.2	0.283
Male	30.2±11.4	35.0±17.1	27.5±5.7	28.0±6.2	0.130*
Female	18.8±5.4	18.9±5.6	19.3±5.0	18.1±5.6	0.612
Time to stand up and sit down, seconds^‡^	20.3±8.4	22.1±10.4	19.2±7.6	19.6±6.7	0.451*

Results expressed are mean (± standard deviation), n (%).

Different letters have different means from multiple comparisons.

* p value: descriptive level of χ^2^ test or Kruskal-Wallis/analysis of variance; ^†^ n=143 (male/female: 38/105); ^‡^ n=136 (of 140 tested, 136 fulfilled the task).

FGF23: fibroblast growth factor 23; 25(OH) vit D: 25(OH) vitamin D; PTH: parathyroid hormone; eGFR: estimated glomerular filtration rate; GDS: Geriatric Depression Scale; MMSE: Mini-Mental State Examination; VFT: Verbal Fluency Test; IADL: Instrumental Activity Daily Living.

There was difference in age, number of chronic diseases and number of regular-use medications in the FGF23’s tertiles. The group with higher FGF23 was slightly older than that in the second tertile (p=0.011). Those with lower mean number of diseases and less use of medications were in the first tertile (p=0.018 and 0.008, respectively). Creatinine had the lowest mean value in the groups with lower FGF23 (p<0.007) and there was an increasing serum cystatin C as the FGF23 increased (p<0.001). The eGFR was higher in the first tertile (p<0.001). The distribution of participants with eGFR <45mL/minute/1.73m^2^was different in the FGF23 tertiles, with the highest proportion of participants in the third tertile and the lowest in the first tertile (p<0.001). Higher mean hemoglobin levels were found in the first tertile of FGF23.

Functional capacity showed no difference between FGF23 tertiles. Since there was a different performance by gender in handgrip strength (mean for male of 30.2kgf, SD ±11.4, and for female of 18.8kgf, SD ±5.4; p=0.0001), we assessed handgrip strength by gender in FGF23 tertiles and also there was no significant association.

According to table 2, in the group with high eGFR (≥45mL/minute/1.73m^2^), the mean FGF23 in the group with lower 25(OH) vitamin D level (<20ng/mL) was higher than the group with 25(OH) vitamin D above 20ng/mL. The multiple linear regression model was fitted to evaluate the simultaneous effects of age, color, number of diseases and medications, 25(OH) vitamin D, eGFR, hemoglobin, creatinine, cystatin C, PTH and phosphorus on FGF23. Considering there were indications of distinct FGF23 behaviors by levels of Vitamin D and eGFR (Table 2), we included the interaction between 25(OH) vitamin D and eGFR in the model. The results of the initial and final regression models are shown in table 3. Only the cystatin C remained significant in the final model (p=0.001). Thus, for an increase of 1mg/L cystatin C, on average, there is an increase of 83.71RU/mL in FGF23.

**Table 2:** Summarized measures of fibroblast growth factor 23 levels according to estimated glomerular filtration rate and 25 OH vitamin D

	eGFR <45mL/minute/1.73m^2^		eGFR ≥45mL/minute/1.73m^2^		p value
	25(OH)D <20ng/mL	25(OH)D ≥20ng/mL	25(OH)D <20ng/mL	25(OH)D ≥20ng/mL	
FGF23 (RU/mL)					<0.001
Mean±SD	138.6±93.1	109.2±84.0	75.6±42.8^a^	68.5±41.7^b^	
95%CI	104.4-172.7	66.00-152.4	63.9-87.3	55.5-81.5	
n (%)	31 (21.5)	17 (11.8)	54 (37.5)	42 (29.2)	

p value: descriptive level of Kruskal-Wallis test. Kolmogorov-Smirnov test (p<0.001). Different letters have different means from multiple comparisons. eGFR: estimated glomerular filtration rate; 25(OH)D: 25(OH) vitamin D; FGF23: fibroblast growth factor 23; SD: standard deviation; 95%IC: 95% confidence interval.

**Table 3:** Estimates of the coefficients and respective 95% confidence interval of the multiple linear regression models on fibroblast growth factor 23 levels

	Initial model		Final model	
	Coefficient (95%CI)	p value	Coefficient (95%CI)	p value
Age, years	-0.80 (-3.62-2.03)	0.578		
Non-white	-10.66 (-34.03-12.72)	0.369		
Number of chronic diseases	-0.25 (-7.04-6.54)	0.942		
Number of regular-use medications	3.31 (-1.34-7.97)	0.161		
25(OH)D <20ng/mL	6.49 (-20.14-33.12)	0.631		
eGFR <45mL/minute/1.73m^2^	5.48 (-36.25-47.21)	0.795		
25(OH)D*eGFR	7.76 (-39.2-54.73)	0.744		
Hemoglobin, g/dL	-3.32 (-12.46-5.83)	0.474		
Creatinine, mg/dL	-15.36 (-71.44-40.72)	0.589		
Cystatin C, mg/L	71.06 (15.23-126.9)	0.013	84.19 (57.56-110.82)	<0.001
PTH, pg/mL	0.13 (-0.22-0.47)	0.477		
Phosphorus	13.49 (-6.59-33.56)	0.186		
R^2^, %	28.8		21.6	
n (%)	134		144	

Kolmogorov-Smirnov: initial (p<0.001) and final model (p<0.001). 25(OH)D*eGFR: interaction between 25(OH)D and eGFR in classes. 95%CI: 95% confidence interval; 25(OH)D: 25(OH) vitamin D; eGFR: estimated glomerular filtration rate; PTH: parathyroid hormone; R^2^: Coefficient of determination.

## DISCUSSION

Serum FGF23 was associated with neither cognitive function nor physical performance in independent individuals aged 80 years or older. We found that those elderly with higher levels of FGF23 were older, had lower hemoglobin levels, more comorbidities, polypharmacy and greater impairment of renal function. These variables can estimate the general health status of the elderly and, thus, suggest that FGF23 may be a biomarker of aging, but FGF23 fails as a biomarker in the functional perspective of healthy aging.

A study of 2,977 elderly people, with an average age of 78 years,^(18,19)^ showed that serum levels of FGF23 were correlated with general health status as found in our study; in addition, the authors also showed the association of FGF23 with the frailty phenotype. However, when each component of frailty measured by physical performance was assessed individually, it was found that FGF23 duplication was associated with a 22% higher probability of slowness measured by the gait speed test, but no correlation with measured weakness by the grip strength in accordance with our result. This group of elderly people was younger and had a higher eTFG than ours, which reflects a lower serum level of FGF23 in this group. It is possible that there is a counterpoint between the increase in FGF23 due to the decline in renal function with aging or due to a healthy lifestyle, with a higher rate of physical activity.^(8)^ None of these analyses addressed possible endogenous stimuli, such as regular physical exercise.

Our population had a good cognitive performance and ability to perform daily tasks for community life (AIVD score). The homogeneity of voluntary participants made it difficult to show these associations with FGF23. However, physical performance was more diverse. Our population performed well on average in upper limbs, as clinically relevant weakness is the handgrip strength of less than 26kgf for men and 16kgf for women.^(19)^ But for the lower limbs, our patients performed poorly, on average, in the timed ability to rise from a chair, over 16.7 seconds.^(16)^ In both measures, there is a relevant intragroup heterogeneity.

In older community-dwelling adults, common aging features were associated with FGF23, such as reduced eGFR and CKD, left ventricular hypertrophy, cardiac insufficiency, cardiovascular disease, increased fat mass, dyslipidemia and increased mortality.^(20-24)^ In this study, patients with cardiovascular disease were excluded. Our results show that the serum FGF23 was related to renal function impairment. For example, among those with eGFR <45mL/minute/1.73m^2^, FGF23 levels were significantly higher. It is in accordance with FGF23 as an early biomarker for kidney dysfunction.^(20,25)^ Another key index in renal function was the association between cystatin C and FGF23. For each 1mg/L increase in cystatin C, there was an increase of approximately 85 units of FGF23. This is in accordance with the idea that cystatin C is a better biomarker of renal function for octogenarians than creatinine because cystatin C is unaffected by muscle mass and dietary protein intake. As FGF23 participates in the regulation of phosphate and the vitamin D metabolism, we expected to find association with them. Among those with eGFR above 45mL/minute/1.73m^2^, there was a correlation between lower levels of 25(OH) vitamin D and higher levels of serum FGF23.

Our study participants are a homogeneous population with multiple comorbidities, but with a capacity for preserved self-care and only light self-reported dependency for tasks related to community life. Although we did not assess nutritional status in detail, BMI was in adequate condition. Their homogeneity could explain our difficulty to find associations between FGF23 and the physical and mental aspects investigated. On the other hand, our approach in assessing different domains of functional capacity pointed some divergence in intra-group physical capacity. The relatively small sample size may have limited the power of the study to detect such associations. In addition, sample size compromised our ability to perform some subgroup analyses (especially in men). It was also not possible to establish a cut-off point below which eGFR could predictably be associated with a rise in FGF23. Other factors, not addressed in this study, especially regular physical activity, may have influenced the results and should be investigated in further studies. Despite the difficulty in estimating GFR in the elderly, we have used the CKD EPI-creatinine-cystatin C equation previously validated in 95 individuals in this cohort.^(26)^

## CONCLUSION

Fibroblast growth factor 23 was not significantly associated with functional performance in community-dwelling independent elderly individuals aged 80 years or more; nonetheless fibroblast growth factor 23 was associated with age, comorbidities, medications and estimated glomerular filtration rate. To our knowledge, this was the first study to evaluate the association between fibroblast growth factor 23 and functional performance among independent elderly individuals.
