# Supplementary material for: Association between fibroblast growth factor 23 and functional capacity among independent elderly individuals
Source: Einstein (Sao Paulo). 2021 Jul 22;19:eAO5925. doi: 10.31744/einstein_journal/2021AO5925 (PMC8302224; doi:10.31744/einstein_journal/2021AO5925)
Supplement: Translation

# Translation: Associação entre fator de crescimento de fibroblastos 23 e capacidade funcional em idosos independentes

**Authors:** Lara Miguel Quirino Araújo, Patrícia Ferreira do Prado Moreira, Clineu de Mello Almada, Luciano Vieira de Araújo, Aline Granja Costa, Ricardo de Castro Cintra Sesso, John P Bilezikian, Marise Lazaretti-Castro, Maysa Seabra Cendoroglo

**Affiliations:** 1 Brasil Escola Paulista de Medicina, Universidade Federal de São Paulo, São Paulo, SP, Brasil.; 2 Brasil Escola de Artes, Ciências e Humanidades, Universidade de São Paulo, São Paulo, SP, Brasil.; 3 United States Department of Endocrinology, College of Physicians and Surgeons, Columbia University, Nova Iorque, NY, United States.

**Keywords:** Fatores de crescimento de fibroblastos, Estado funcional, Envelhecimento cognitivo, Envelhecimento, Idoso de 80 anos ou mais, Rim/fisiologia, Atividades cotidianas, Sarcopenia

## Abstract

**Objetivo:**

Examinar a associação entre o fator de crescimento de fibroblastos 23 sérico e a capacidade funcional em indivíduos independentes, com 80 anos ou mais.

**Métodos:**

A capacidade funcional de 144 idosos foi avaliada por meio de Atividades Instrumentais da Vida Diária, testes cognitivos, força de preensão manual e capacidade de levantar de uma cadeira e sentar cinco vezes. O fator de crescimento de fibroblastos 23 foi medido pelo teste ELISA.

**Resultados:**

Os participantes no tercil mais baixo de fator de crescimento de fibroblastos 23 tiveram a maior média±desvio-padrão da taxa de filtração glomerular estimada, concentração média de hemoglobina mais alta, menor número médio de doenças e menor número de medicamentos utilizados. Em participantes com taxa de filtração glomerular estimada >45mL/minuto/1,73m^2^, o nível médio do fator de crescimento de fibroblastos 23 foi maior naqueles com 25(OH) vitamina D <20ng/mL do que naqueles com 25(OH) vitamina D ≥20ng/mL (75,6RU/mL±42,8 *versu*s 68,5RU/mL±41,7; p<0,001). Houve aumento na cistatina C sérica média (de 1,3mg/mL±0,3 a 1,5mg/mL±0,3 a 1,7mg/mL±0,4) em função do tercil de fator de crescimento 23 de fibroblastos mais alto (p<0,001). Os níveis de fator de crescimento de fibroblastos 23 não foram significativamente associados à capacidade em testes físicos ou cognitivos.

**Conclusão:**

Em idosos independentes residentes na comunidade ≥80 anos, o fator de crescimento de fibroblastos 23 foi associado a comorbidades relacionadas à idade e à função renal, mas não à capacidade funcional.

## INTRODUÇÃO

A identificação de biomarcadores do processo de envelhecimento pode potencialmente promover o desenvolvimento de alvos para a prevenção de síndromes associadas ao envelhecimento. O fator de crescimento de fibroblastos 23 (FGF23) é um alvo candidato, porque é uma proteína-chave nas vias bioquímicas associadas ao envelhecimento. Os eixos endócrinos FGF-Klotho têm papel decisivo nas doenças relacionadas ao envelhecimento. O FGF23 é uma proteína endócrina que entra na circulação sistêmica, e a proteína Klotho é o correceptor para se ligar ao FGF23, com alta afinidade ao receptor do fator de crescimento de fibroblastos (FGFR) em seus órgãos-alvo. Os rins são o principal órgão-alvo do FGF23, no qual ocorre a indução à fosfatúria por ação na borda em escova da membrana apical das células tubulares proximais. Além disso, o FGF23, a vitamina D e o hormônio da paratireoide (PTH - *parathyroid hormone*) estão em ciclos de *feedback* negativo para manter a homeostase do fosfato.^(1)^

A falta de FGF23 ou Klotho causa um fenótipo de envelhecimento prematuro, como hipogonadismo, involução tímica prematura, calcificação ectópica, atrofia dérmica, enfisema pulmonar, neurodegeneração, perda auditiva e calcificações vasculares.^(2-5)^ Existem algumas evidências para apoiar esse fenótipo de envelhecimento, com base na hiperfosfatemia.^(6)^ Na doença renal crônica (DRC) e no rim envelhecido, o nível sérico de FGF23 aumenta, para controlar a hiperfosfatemia por meio do eixo FGF23-Klotho-vitamina D-PTH. No entanto, o FGF23 não consegue manter o equilíbrio do fosfato, o que resulta em hiperfosfatasemia, e o fosfato passa a exercer suas ações deletérias. Por outro lado, um experimento com células-tronco mesenquimais de músculo humano, importante na regeneração da massa muscular, mostrou que o tratamento com FGF23 promove o fenótipo de envelhecimento independente do Klotho, por meio de ação direta na regulação do ciclo celular pela proteína p53.^(7)^ Paradoxalmente, um experimento com protocolos de exercício físico em camundongos demonstrou aumento no FGF23 sérico e no mRNA de FGF23 regulado positivamente para exercícios de longo prazo e FGF23 no músculo esquelético. Os camundongos previamente tratados com FGF23 tiveram melhor desempenho em exercícios, controle de espécies reativas de oxigênio (ROS) e melhora da função mitocondrial no músculo esquelético. O mecanismo dessas ações é desconhecido.^(8)^ Esses estudos sugerem que a influência do FGF23 na biologia muscular e no desempenho físico depende do contexto e das diferentes vias. O *deficit* de FGF23 no soro causa envelhecimento prematuro pela perda do controle do fosfato, e a sobrecarga de FGF23 pode levar à senescência celular por p53. Seria possível especular que o envelhecimento acelerado na DRC pode ser influenciado por ambos os mecanismos, dependendo do estágio da DRC. Por outro lado, o FGF23 sérico elevado por um estímulo saudável, como o exercício físico, pode melhorar o desempenho no exercício físico.

A saúde do idoso deve ser entendida a partir de uma perspectiva funcional (e não baseada na doença), uma vez que o envelhecimento saudável é “o processo de desenvolvimento e manutenção da capacidade funcional que permite o bem-estar na idade avançada”. A capacidade funcional significa “os atributos relacionados à saúde que permitem que as pessoas sejam ou façam aquilo que valorizam”.^(9)^ A função cognitiva e o desempenho físico são os pilares desses atributos, e o FGF23 pode influenciar em ambos. Uma amostra do *Framingham Heart Study* mostrou haver associação entre os níveis séricos de FGF23 e a incidência de demência,^(10)^ e que as ações do FGF23 na biologia muscular e no envelhecimento prematuro podem desempenhar papel no desempenho físico.

## OBJETIVO

Examinar a associação entre fator de crescimento de fibroblastos 23 sérico e capacidade funcional em indivíduos independentes com 80 anos ou mais, além de verificar a associação do fator de crescimento de fibroblastos 23 com medições laboratoriais do eixo rim-vitamina D-hormônio da paratireoide.

## MÉTODOS

### Participantes do estudo

Este é um estudo transversal de dados da primeira onda do Projeto Longevidade e incluiu uma coorte de idosos residentes na comunidade com 80 anos ou mais, independentes para Atividades da Vida Diária^(11)^ e recrutados por meio de anúncio em jornais de bairro, rádios ou encaminhamento de outros departamentos da Universidade Federal de São Paulo (UNIFESP) para a Divisão de Geriatria, no período de 2010 a 2012. Os critérios de inclusão foram ser capaz de andar sem apoio humano, ter todas as doenças crônicas sob controle no momento do estudo e não ter queixa cognitiva. Os critérios de exclusão foram morar em casa de repouso; apresentar deficiência cognitiva aparente; ter doença terminal; ser incapaz de responder aos testes do estudo; ter infecção aparente, insuficiência cardíaca, cirrose hepática ou história de diálise; ter passado por terapia imunossupressora nos 6 meses anteriores ou ter se submetido a quimioterapia para câncer, ou ter infecção por HIV. Todos assinaram o Termo de Consentimento Livre e Esclarecido, e o Comitê de Ética em Pesquisa da UNIFESP aprovou o protocolo de estudo 1532/09.

### Medidas

Foram coletados dados sociodemográficos e índice de massa corporal (IMC), dados clínicos, lista de medicamentos e domínios da capacidade funcional por meio de um questionário padrão administrado por médicos treinados.

### Capacidade funcional

Os sintomas depressivos foram obtidos por meio da Escala de Depressão Geriátrica de 15 itens.^(12)^ A função cognitiva foi avaliada pelo Miniexame do Estado Mental (MEEM)^(13)^ e pelo Teste de Fluência Verbal (TFV) de categorias.^(14)^ O MEEM é um teste de cognição global, e o TFV verifica a linguagem, as funções executivas e a memória semântica. A capacidade autorrelatada de realizar tarefas para a vida na comunidade foi avaliada por meio da escala de Atividades Instrumentais de Vida Diária (AIVD) de Lawton, com pontuação de sete a 21 (a pontuação mais alta significa independência).^(15)^O desempenho físico foi avaliado pela habilidade cronometrada para se levantar de uma cadeira e se sentar cinco vezes,^(16)^ bem como pela força de preensão pelo melhor resultado em três tentativas usando um dinamômetro Jamar.^(17)^

### Medidas laboratoriais

As medidas antropométricas e de desempenho foram realizadas ao mesmo tempo, e a coleta de sangue foi feita nos 4 meses subsequentes. As amostras de sangue foram coletadas após jejum de 12 horas, sendo medidos os níveis de creatinina, cistatina C, hemoglobina, albumina, 25(OH) vitamina D sérica e PTH intacto. O FGF23 sérico foi analisado por um *kit* de segunda geração do *Enzyme Linked Immunonosorbent Assay* (ELISA) de dois locais para terminal C de FGF23 (Immutopics, San Clemente, CA, Estados Unidos). Esse teste detecta dois epítopos no terminal C de FGF23 e tem sensibilidade de 1,5 unidade relativas por mL (RU/mL) e coeficientes de variação inter- e intraensaio de menos de 5%. A creatinina sérica foi medida usando um método colorimétrico de Jaffé cinético modificado em um analisador automático (AU 400, Beckman Coulter, CA, Estados Unidos), que foi calibrado para espectrometria de massa de diluição isotópica, usando material de referência padrão (914a) rastreável para o *National Institutes of Standards and Technology* (NIST). Os níveis plasmáticos de cistatina C foram determinados por método imunoturbidimétrico intensificado por partículas automatizado usando um analisador Beckman AU 400 (Beckman Coulter) e reagentes (códigos LX002. s2361. X0973. X0974) obtidos de DakoCytomation (Glostrup, Dinamarca), seguindo os procedimentos recomendados pelo fabricante dos reagentes. Calculou-se a estimativa da taxa de filtração glomerular (eTFG) pela equação CKD-EPI creatinina-cistatina C. Todas as amostras foram congeladas a -80° C.

### Análise dos dados

As análises foram realizadas no (SPSS) versão 20.0. Os níveis de FGF23 foram categorizados em tercis e examinou-se a distribuição das covariáveis em suas categorias. Os domínios da capacidade funcional foram apresentados como média (± desvio-padrão – DP). O teste do χ^2^ foi usados para examinar as associações entre as variáveis categóricas; a análise de variância (Anova) foi usada para comparar as médias de mais de dois grupos; o teste de Duncan ou o teste C de Dunnett foi utilizado para comparações múltiplas; o teste de Kolmogorov-Smirnov e o teste de Levene foram utilizados para verificar a normalidade e a homoscedasticidade, respectivamente; a correção de Brown-Forsythe foi empregada no caso de presunção errônea de homoscedasticidade para corrigir os graus de liberdade da estatística F; o teste de Kruskal-Wallis foi utilizado nos casos de distribuição não paramétrica; e, caso o teste de Kruskal-Wallis fosse significativo, a diferença entre os grupos foi avaliada pelo teste de Bonferroni-Dunn. Na regressão linear múltipla, as características clínicas foram variáveis independentes, e o FGF23 foi uma variável dependente. Inicialmente todas as variáveis foram incluídas no modelo e, em seguida, os 5% não significativos foram excluídos um a um, em ordem de significância (método *backward*).

## RESULTADOS

Foram estudados 144 pacientes, com média de idade de 85,4 anos, 73,6% eram mulheres, com média de quatro doenças crônicas e uso regular de seis medicamentos. O FGF23 geral foi de 91,05RU/mL±67,47. Foi observada eTFG menor que 45mL/minuto/1,73m^2^ em 33,3%, e 25(OH) vitamina D <20ng/mL estava presente em 59% de todos os participantes. A tabela 1 mostra todos os participantes agrupados por tercis do FGF23 para investigar diferenças demográficas, clínicas, laboratoriais e de capacidade funcional.

**Tabela 1:** Parâmetros demográficos, clínicos e laboratoriais da população do estudo por tercis de níveis de fator de crescimento de fibroblastos 23

Características	Todos (n=144)	FGF23 (RU/mL)			Valor de p
		1^o^ tercil <54,4 (n=47)	2^o^ tercil 54,4-92,6 (n=48)	3^o^ tercil ≥92,6 (n=49)	
Idade, anos	85,4±4,1	85,6±3,8	84,1±3,0^a^	86,6±4,9^b^	0,011
Sexo					0,931
Masculino	38 (26,4)	13 (27,7)	13 (27,1)	12 (24,5)	
Feminino	106 (73,6)	34 (72,3)	35 (72,9)	37 (75,5)	
Raça					0,069
Branca	96 (66,7)	26 (55,3)	32 (66,7)	38 (77,6)	
Não branca	48 (33,3)	21 (44,7)	16 (33,3)	11 (22,4)	
Número de doenças crônicas	4,4±1,9	3,9±1,9^a^	4,5±1,6	4,9±2,0^b^	0,018
Número de medicamentos de uso regular	5,9±2,7	4,9±2,1^a^	6,2±2,6^b^	6,5±3,1^b^	0,008
Suplementos de vitamina D	59 (41,3)	15 (31,9)	23 (47,9)	21 (43,8)	0,260
Peso, kg	70,7±78,9	65,1±12,5	82,4±135,6	64,7±13,8	0,687
Índice de massa corporal, kg/m^2^	26,8±4,3	27,4±4,0	26,5±4,1	26,5±4,9	0,562
25(OH)D, ng/mL	19,2±8,4	19,6±8,6	20,8±9,6	17,3±6,4	0,110
25(OH)D – categoria, ng/mL					0,183
<20ng/mL	85 (59,0)	26 (55,3)	25 (52,1)	34 (69,4)	
≥20ng/mL	59 (41,0)	21 (44,7)	23 (47,9)	15 (30,6)	
PTH, pg/mL	62,6±33,4	53,9±29,2	63,1±27,8	70,5±40,0	0,060*
Cálcio, mg/dL	9,3±0,5	9,3±0,5	9,4±0,4	9,4±0,5	0,428
Fósforo, mg/dL	3,4±1,1	3,2±0,5	3,3±0,4	3,7±1,8	0,091*
Hemoglobina, g/dL	13,4±1,3	13,9±1,2^b^	13,3±1,3^a^	13,1±1,3^a^	0,012
Creatinina, mg/dL	0,96±0,28	0,87±0,19^a^	0,97±0,24^b^	1,04±0,35^b^	0,007
Cistatina C, mg/L	1,50±0,37	1,32±0,28^c^	1,49±0,29^a^	1,69±0,43^b^	<0,001
eTFG, mL/minuto/1,73m^2^	51±14	58±12^b^	50±12^a^	45±14^a^	<0,001
eTFG – categoria, mL/minuto/1,73m^2^					<0,001
<45mL/minuto/1,73m^2^	48 (33,3)	6 (12,8)^a^	17 (35,4)	25 (51,0)^b^	
≥45mL/minuto/1,73m^2^	96 (66,7)	41 (87,2)	31 (64,6)	24 (49,0)	
Capacidade funcional					
Educação, anos	4,5±3,8	4,4±3,7	5,1±4,6	3,9±2,9	0,720*
EDG	3,9±2,7	3,4±2,2	3,9±2,9	4,4±2,8	0,259*
MMSE	24,2±3,4	23,7±3,2	24,5±3,6	24,5±3,3	0,466
TFV	12,9±3,5	12,5±3,4	12,9±3,6	13,3±3,7	0,562
AIVD	18,8±2,5	19,0±2,6	19,3±2,1	18,2±2,8	0,156*
Força de preensão manual, kgf^†^					
Todos	21,8±9,0	23,5±12,4	21,6±6,3	20,5±7,2	0,283
Masculino	30,2±11,4	35,0±17,1	27,5±5,7	28,0±6,2	0,130*
Feminino	18,8±5,4	18,9±5,6	19,3±5,0	18,1±5,6	0,612
Tempo para levantar-se e sentar-se (segundos)^‡^	20,3±8,4	22,1±10,4	19,2±7,6	19,6±6,7	0,451*

Resultados expressos como média (± desvio padrão), n (%). Letras sobrescritas diferentes indicam médias diferentes de comparações múltiplas. * Valor de p: nível descritivo do teste χ^2^ ou Kruskal-Wallis/análise de variância; ^†^ n=143 (masculino/feminino: 38/105); ^‡^ n=136 (de 140 testados, 136 cumpriram a tarefa). FGF23: fator de crescimento de fibroblastos 23; 25(OH)D: 25(OH) vitamina D; PTH: hormônio da paratireoide; eTFG: estimativa da taxa de filtração glomerular; EDG: Escala de Depressão Geriátrica; MEEM: Miniexame do Estado Mental; TFV: Teste de Fluência Verbal; AIVD: Atividade Instrumental da Vida Diária.

Houve diferença em idade, número de doenças crônicas e número de medicamentos de uso regular nos tercis do FGF23. O grupo com maior FGF23 era ligeiramente mais velho que o do segundo tercil (p=0,011). Aqueles com menor número médio de doenças e menor uso de medicamentos estavam no primeiro tercil (p=0,018 e 0,008, respectivamente). A creatinina teve o menor valor médio nos grupos com menor FGF23 (p<0,007), e houve aumento da cistatina C sérica à medida que o FGF23 aumentou (p<0,001). A eTFG foi maior no primeiro tercil (p<0,001). A distribuição dos participantes com eTFG <45mL/minuto/1,73m^2^foi diferente nos tercis de FGF23, com a maior proporção de participantes no terceiro tercil e a menor no primeiro tercil (p<0,001). Os níveis médios mais elevados de hemoglobina foram encontrados no primeiro tercil de FGF23.

A capacidade funcional não mostrou diferença entre os tercis de FGF23. Como houve desempenho diferente por gênero na força de preensão manual (média para homens de 30,2kgf, com DP ±11,4, e para mulheres de 18,8kgf, com DP ±5,4; p=0,0001), avaliou-se a força de preensão por gênero nos tercis de FGF23, e também não houve associação significativa.

De acordo com a tabela 2, no grupo com alta eTFG (≥45mL/minuto/1,73m^2^), a média de FGF23 no grupo com menor nível de 25(OH) vitamina D (<20ng/mL) foi maior que no grupo com 25(OH) vitamina D acima de 20ng/mL. O modelo de regressão linear múltipla foi ajustado para avaliar os efeitos simultâneos de idade, cor, número de doenças e medicamentos, 25(OH) vitamina D, eTFG, hemoglobina, creatinina, cistatina C, PTH e fósforo no FGF23. Como havia indicações de comportamentos distintos do FGF23 por níveis de vitamina D e eTFG (Tabela 2), foi incluída a interação entre 25(OH) vitamina D e eTFG no modelo. Os resultados dos modelos de regressão inicial e final são mostrados na tabela 3. Apenas a cistatina C permaneceu significativa no modelo final (p=0,001). Assim, para um aumento de 1mg/L de cistatina C, em média, ocorreu aumento de 83,71RU/mL no FGF23.

**Tabela 2:** Medidas resumidas dos níveis de fator de crescimento de fibroblastos 23 de acordo com a taxa de filtração glomerular estimada e 25(OH) vitamina D

	eTFG <45mL/minuto/1,73m^2^		eTFG ≥45mL/minuto/1,73m^2^		Valor de p
	25(OH)D <20ng/mL	25(OH)D ≥20ng/mL	25(OH)D <20ng/mL	25(OH)D ≥20ng/mL	
FGF23, pg/mL					<0,001
Média ± DP	138,6±93,1	109,2±84,0	75,6±42,8^a^	68,5±41,7^b^	
IC95%	104,4-172,7	66,00-152,4	63,9-87,3	55,5-81,5	
n (%)	31 (21,5)	17 (11,8)	54 (37,5)	42 (29,2)	

Valor de p: nível descritivo do teste de Kruskal-Wallis. Teste de Kolmogorov-Smirnov (p<0,001). Letras sobrescritas diferentes indicam médias diferentes de comparações múltiplas. eTFG: estimativa da taxa de filtração glomerular; 25(OH)D: 25(OH) vitamina D; FGF23: fator de crescimento de fibroblastos 23; DP: desvio-padrão; IC95%: intervalo de confiança de 95%.

**Tabela 3:** Estimativas dos coeficientes e respectivo intervalo de confiança de 95% dos modelos de regressão linear múltipla em níveis de fator de crescimento de fibroblastos 23

	Modelo inicial		Modelo final	
	Coeficiente (IC95%)	Valor de p	Coeficiente (IC95%)	Valor de p
Idade, anos	-0,80 (-3,62-2,03)	0,578		
Não branca	-10,66 (-34,03-12,72)	0,369		
Número de doenças crônicas	-0,25 (-7,04-6,54)	0,942		
Número de medicamentos de uso regular	3,31 (-1,34-7,97)	0,161		
25(OH)D <20ng/mL	6,49 (-20,14-33,12)	0,631		
eTFG <45mL/minuto/1,73m^2^	5,48 (-36,25-47,21)	0,795		
25(OH)D*eTFG	7,76 (-39,2-54,73)	0,744		
Hemoglobina, g/dL	-3,32 (-12,46-5,83)	0,474		
Creatinina, mg/dL	-15,36 (-71,44-40,72)	0,589		
Cistatina C, mg/L	71,06 (15,23-126,9)	0,013	84,19 (57,56-110,82)	<0,001
PTH, pg/mL	0,13 (-0,22-0,47)	0,477		
Fósforo	13,49 (-6,59-33,56)	0,186		
R^2^, %	28,8		21,6	
n (%)	134		144	

Kolmogorov-Smirnov: modelo inicial (p<0,001) e final (p<0,001). 25(OH)D*eTFG: interação entre 25(OH)D e eTFG em classes. IC95%: intervalo de confiança de 95%; 25(OH)D: 25(OH) vitamina D; eTFG: estimativa da taxa de filtração glomerular; PTH: hormônio da paratireoide; R^2^: coeficiente de determinação.

## DISCUSSÃO

O FGF23 sérico não foi associado à função cognitiva nem ao desempenho físico em indivíduos independentes com 80 anos ou mais. Ainda, idosos com níveis mais elevados de FGF23 eram mais velhos, tinham níveis mais baixos de hemoglobina, mais comorbidades, polifarmácia e maior comprometimento da função renal. Essas variáveis podem estimar o estado geral de saúde do idoso e, assim, sugerir que o FGF23 pode ser um biomarcador do envelhecimento, mas o FGF23 não serve como biomarcador na perspectiva funcional do envelhecimento saudável.

Um estudo realizado com 2.977 idosos, com média de idade de 78 anos,^(18,19)^ mostrou que os níveis séricos de FGF23 se correlacionaram com o estado geral de saúde, como encontrado neste estudo; além disso, os autores também mostraram a associação do FGF23 com o fenótipo de fragilidade. No entanto, quando cada componente da fragilidade medida pelo desempenho físico foi avaliado individualmente, verificou-se que a duplicação do FGF23 foi associada a uma probabilidade 22% maior de lentidão medida pelo teste de velocidade de marcha, mas sem nenhuma correlação com a fraqueza medida pela força de preensão, de acordo com os presentes resultados. Esse grupo de idosos era mais jovem e tinha eTFG maior do que a deste estudo, o que reflete um nível sérico mais baixo de FGF23 nesse grupo. É possível que haja um contraponto entre o aumento do FGF23 devido ao declínio da função renal com o envelhecimento ou por um estilo de vida saudável, com maior taxa de atividade física.^(8)^ Nenhuma dessas análises abordou possíveis estímulos endógenos, como o exercício físico regular.

A população deste estudo apresentou bom desempenho cognitivo e capacidade de realizar tarefas diárias para a vida comunitária (pontuação das AIVDs). A homogeneidade dos voluntários dificultou mostrar essas associações com o FGF23. Porém, o desempenho físico foi mais diversificado. Nossa população teve bom desempenho em média nos membros superiores, já que a fraqueza clinicamente relevante é a força de preensão manual de menos de 26kgf para homens e 16kgf para mulheres.^(19)^ Porém, para os membros inferiores, nossos pacientes tiveram desempenho ruim, em média, na capacidade cronometrada de se levantar de uma cadeira, com mais de 16,7 segundos.^(16)^ Em ambas as medidas, a heterogeneidade intragrupo é relevante.

Em adultos mais velhos que vivem na comunidade, características comuns de envelhecimento foram associadas ao FGF23, como redução da eTFG e DRC, hipertrofia ventricular esquerda, insuficiência cardíaca, doença cardiovascular, aumento da massa gorda, dislipidemia e aumento da mortalidade.^(20-24)^ Neste estudo, os pacientes com doença cardiovascular foram excluídos. Os presentes resultados mostram que o FGF23 sérico foi relacionado ao comprometimento da função renal. Por exemplo, entre aqueles com eTFG <45mL/minuto/1,73m^2^, os níveis de FGF23 foram significativamente mais elevados. Isso condiz com o FGF23 ser um biomarcador precoce para disfunção renal.^(20,25)^ Outro índice fundamental na função renal foi a associação entre cistatina C e FGF23. Para cada aumento de 1mg/L na cistatina C, houve aumento de aproximadamente 85 unidades de FGF23. Isso condiz com a noção de que a cistatina C é um biomarcador melhor da função renal para octogenários do que a creatinina, porque a cistatina C não é afetada pela massa muscular e pela ingestão de proteína na dieta. Como o FGF23 participa da regulação do fosfato e do metabolismo da vitamina D, esperava-se encontrar associação com eles. Entre aqueles com eTFG acima de 45mL/minuto/1,73m^2^, houve correlação entre os níveis mais baixos de 25(OH) vitamina D e os níveis mais altos de FGF23 sérico.

Os participantes deste estudo formam uma população homogênea com múltiplas comorbidades, mas com capacidade de autocuidado preservada e apenas leve autorrelato de dependência para tarefas relacionadas à vida comunitária. Embora o estado nutricional não tenha sido avaliado em detalhes, o IMC estava em condições adequadas. Sua homogeneidade poderia explicar nossa dificuldade em encontrar associações entre o FGF23 e os aspectos físicos e mentais investigados. Por outro lado, nossa abordagem na avaliação de diferentes domínios da capacidade funcional apontou algumas divergências na capacidade física intragrupo. O tamanho relativamente pequeno da amostra pode ter limitado o poder do estudo para detectar tais associações. Além disso, o tamanho da amostra comprometeu nossa capacidade de realizar algumas análises de subgrupos (especialmente em homens). Também não foi possível estabelecer um ponto de corte abaixo do qual a eTFG poderia estar previsivelmente associada a um aumento no FGF23. Outros fatores, não abordados neste estudo, principalmente a prática de atividade física regular, podem ter influenciado nos resultados e devem ser investigados em estudos futuros. Apesar da dificuldade em estimar a TFG em idosos, usamos a equação CKD EPI-creatinina-cistatina C previamente validada em 95 indivíduos desta coorte.^(26)^

## CONCLUSÃO

O fator de crescimento de fibroblastos 23 não foi significativamente associado ao desempenho funcional em idosos independentes residentes na comunidade com 80 anos ou mais, mas esteve associado com idade, comorbidades, medicamentos e taxa de filtração glomerular estimada. Até onde se sabe, este foi o primeiro estudo a avaliar a associação entre o fator de crescimento de fibroblastos 23 e o desempenho funcional em idosos independentes.
